# ROS-Dependent Activation of Autophagy through the PI3K/Akt/mTOR Pathway Is Induced by Hydroxysafflor Yellow A-Sonodynamic Therapy in THP-1 Macrophages

**DOI:** 10.1155/2017/8519169

**Published:** 2017-01-16

**Authors:** Yueqing Jiang, Jiayuan Kou, Xiaobo Han, Xuesong Li, Zhaoyu Zhong, Zhongni Liu, Yinghong Zheng, Ye Tian, Liming Yang

**Affiliations:** ^1^Department of Pathophysiology, Key Laboratory of Cardiovascular Pathophysiology, Harbin Medical University, Harbin, China; ^2^Division of Cardiology, The First Affiliated Hospital, Harbin Medical University, Harbin, China

## Abstract

Monocyte-derived macrophages participate in infaust inflammatory responses by secreting various types of proinflammatory factors, resulting in further inflammatory reactions in atherosclerotic plaques. Autophagy plays an important role in inhibiting inflammation; thus, increasing autophagy may be a therapeutic strategy for atherosclerosis. In the present study, hydroxysafflor yellow A-mediated sonodynamic therapy was used to induce autophagy and inhibit inflammation in THP-1 macrophages. Following hydroxysafflor yellow A-mediated sonodynamic therapy, autophagy was induced as shown by the conversion of LC3-II/LC3-I, increased expression of beclin 1, degradation of p62, and the formation of autophagic vacuoles. In addition, inflammatory factors were inhibited. These effects were blocked by Atg5 siRNA, the autophagy inhibitor 3-methyladenine, and the reactive oxygen species scavenger N-acetyl cysteine. Moreover, AKT phosphorylation at Ser473 and mTOR phosphorylation at Ser2448 decreased significantly after HSYA-SDT. These effects were inhibited by the PI3K inhibitor LY294002, the AKT inhibitor triciribine, the mTOR inhibitor rapamycin, mTOR siRNA, and N-acetyl cysteine. Our results demonstrate that HSYA-SDT induces an autophagic response via the PI3K/Akt/mTOR signaling pathway and inhibits inflammation by reactive oxygen species in THP-1 macrophages.

## 1. Introduction

Atherosclerosis is a common cardiovascular disease linked to inflammation and is one of the most serious threats to human health [1–3]. Macrophages accumulate in atherosclerotic plaques and secrete various proinflammatory factors such as IL-1, IL-12, and tumor necrosis factor, resulting in further inflammatory reactions in the lesions and even rupture of the plaques [4–6]. Reducing the inflammatory infiltration of macrophages in the plaques would slow or prevent atherosclerosis progression and enhance plaque stability [7].

Autophagy is a conserved, life-sustaining response to cellular stress conditions [8, 9]. A growing body of evidence has shown that autophagy plays a critical role in modulating atherogenesis and enhancing atherosclerotic plaque stability [10, 11], a major mechanism underlying autophagy-mediated inhibition of inflammation [12, 13]. Therefore, induction of autophagy may be a potential treatment for atherosclerosis. Mammalian target of rapamycin (mTOR) plays a crucial role in cell survival and is a key regulator of autophagy [14–16].

Interventional therapy for atherosclerosis is still the major choice among clinical patients. Nevertheless, it has some side effects, such as poor selectivity of interventional tools [17] and possible postoperative restenosis. Sonodynamic therapy (SDT) is a novel noninvasive approach [18] based on a combination of low-intensity ultrasound and a sensitizing drug (sonosensitizer) to rapidly produce biological changes among neighboring cells. An initial clinical application of SDT showed therapeutic benefits in cancer patients [19]. Moreover, our group demonstrated that SDT had a beneficial effect on atherosclerosis as it could induce apoptosis of macrophages in vitro [20–22] and effectively stabilized the atherosclerotic plaques of the rabbit femoral artery [23]. Reactive oxygen species (ROS) were found to be the most important factors in the biological effects induced by SDT in these studies. Many reports have suggested that ROS are classical autophagy inducers [24, 25], and correspondingly, autophagy was enhanced after SDT treatment in several cell types [26–28].

The application of sonosensitizers is one of the most critical factors for SDT. In recent years, our group has identified various sonosensitizers derived from the extracts of Chinese herbs for future clinical use [20, 21, 29]. Hydroxysafflor yellow A (HSYA) is a major constituent of the hydrophilic fraction of the safflower plant, a traditional Chinese herbal medicine with a long history of clinical treatment of cardiovascular diseases by intravenous injection [30]. Satisfactory water-solubility and high safety of HSYA [31] prompted us to explore its sonosensitivity. In this study, we investigated whether HSYA could be used as a sonosensitizer and whether the combination of HSYA and ultrasound (HSYA-SDT) could induce autophagy and inhibit inflammation via the PI3K/Akt/mTOR pathway by ROS generation in THP-1 macrophages.

## 2. Materials and Methods

### 2.1. Cell Culture

Human THP-1 monocytes (American Type Culture Collection, Manassas, VA, USA) were cultured in RPMI 1640 medium (HyClone, Logan, UT, USA) supplemented with 10% fetal bovine serum (FBS, HyClone, Logan, UT, USA), 20 *μ*g/mL penicillin, and 20 *μ*g/mL streptomycin (Sigma-Aldrich Co., St. Louis, MO, USA). The cells were maintained at 37°C in a humidified incubator with 5% CO_2_, and the medium was refreshed every 2-3 days. For experiments, the cells were seeded in 35 mm Petri dishes or 96-well plates at a density of 1.0 × 10^5^ cells per milliliter and were differentiated into macrophages by adding 100 ng/mL phorbol-12-myristate-13-acetate (PMA, EMD Biosciences Inc., La Jolla, CA, USA) for 72 h. All of the studies were carried out using complete medium to avoid the activation of autophagy by starvation.

### 2.2. Ultrasound Exposure System

The ultrasound exposure system used in this study was assembled by Condensed Matter Science and Technology Institute of the Harbin Institute of Technology (Harbin, China), as previously described [21].

### 2.3. SDT Protocol

HSYA was obtained from Chengdu Must Bio-Technology Co., Ltd., and was stored in ddH_2_O as a 5 mmol/L stock solution at 4°C in the dark. Working solutions were freshly diluted to different concentrations by 1640 medium containing 10% FBS.

THP-1 derived macrophages were collected, differentiated, and randomly divided into four groups: (1) Control, (2) ultrasound alone, (3) HSYA alone, and (4) HSYA with ultrasound (HSYA-SDT). For the HSYA and HSYA-SDT groups, the cells were incubated with the indicated doses of HSYA for a drug loading time of 4 h in FBS-loaded RPMI 1640 medium. For the Control and ultrasound alone groups, an equivalent volume of medium was used instead of HSYA. The cells in the ultrasound and HSYA-SDT groups were exposed to ultrasound at a frequency of 1.0 MHz and at the indicated intensities and times. After the treatments, the cells were carefully washed once in phosphate-buffered saline (PBS), cultured in fresh medium for several minutes, and then prepared for different analyses.

Based on the different types of inhibitory analyses, 10 mM of the autophagy inhibitor 3-methyladenine (3-MA, Sigma-Aldrich Co., St. Louis, MO, USA), 100 nM of the autophagy inhibitor bafilomycin A1 (ba A1, Sigma-Aldrich Co., St. Louis, MO, USA), 50 *μ*M of the autophagy inhibitor hydroxychloroquine (Selleck Chemicals, Houston, TX, USA), 5 *μ*M of the AKT inhibitor triciribine (Selleck Chemicals, Houston, TX, USA), 5 *μ*M of the PI3K agonist insulin-like growth factors-1 (IGF-1, R&D Systems, Inc., Minneapolis, USA), 5 *μ*M of the PI3K inhibitor LY294002 (Selleck Chemicals, Houston, TX, USA), and 1 *μ*M of the mTOR inhibitor rapamycin (Rapa, Selleck Chemicals, Houston, TX, USA) were added to the culture medium together with HSYA for 4 h. Additionally, 1 mM of the ROS scavenger N-acetyl-cysteine (NAC) was added 30 min before HSYA-SDT.

### 2.4. Cell Viability Assay

Cell survival was detected using CCK-8 assays (Beyotime Biotechnology, Inc., Beijing, China). THP-1 macrophages were seeded in 96-well culture plates, and after treatment, the plate was carefully washed once in PBS at the indicated time. Then, 100 *μ*L of medium containing CCK-8 was added to each well (the ratio of medium and CCK-8 volume was 9 : 1). After the plates were incubated for 30 min at 37°C in the dark, the absorption at 450 nm of each well was measured using a microplate reader (Varian Australia Pty Ltd., Australia). The data are shown as the average of five wells for each group.

### 2.5. Detection of Intracellular ROS

Intracellular ROS measurement was performed by measuring the fluorescence intensity of 2′,7′-dichlorofluorescein (DCF). The cells were washed with PBS and then stained with 20 *μ*M dichlorodihydrofluorescein diacetate (DCFH-DA, Applygen Technologies Co., Ltd., Beijing, China) for 15 min at 37°C in the dark at the indicated time after HSYA-SDT. Then, the cells were washed twice with PBS. For flow cytometry analysis, the cells were harvested, and the fluorescence signals produced were analyzed by using a FACSVerse flow cytometer (BD, Germany). Fluorescence was measured with a fluorescence microscope (Olympus IX81, Japan) at 488 nm excitation and 525 nm emission wavelengths, and photos were taken.

### 2.6. Transmission Electron Microscopy Examination

At 30 min after HSYA-SDT, the cells were harvested by centrifugation and analyzed as previously described [20]. The images were observed and obtained with a transmission electron microscope (JEM-1220, Japan).

### 2.7. Western Blotting Assay

At the indicated times after treatment, RIPA lysis buffer was used to extract the total cellular proteins on ice. The protein concentration was detected using a bicinchoninic acid (BCA) kit (Beyotime Biotechnology, Inc., Beijing, China). Denatured protein samples were separated using sodium dodecyl sulfate-polyacrylamide gel electrophoresis (SDS-PAGE) and transferred onto 0.45 *μ*m PVDF membranes (Millipore, Schwalbach, Germany) at 300 mA. After the membranes were blocked at room temperature for 1 h in blocking buffer containing 5% dried skim milk diluted with Tris-buffered saline-Tween 20 (TBST), they were incubated with primary antibodies against LC3B (Sigma-Aldrich Co., St. Louis, MO, USA), p62, mTOR, p-mTOR, AKT, p-AKT, Atg5 (Cell Signaling Technology, Inc., USA), beclin 1, IL-1*β*, IL-12, TNF-*α* (Abcam, Burlingame, CA, USA), and *β*-actin (ZSGB-BIO, Inc., Beijing, China, all primary antibodies above were diluted 1 : 1000) at 4°C overnight. After the membranes were washed with TBST, they were incubated with HRP-conjugated secondary antibodies (ZSGB-BIO, Inc., Beijing, China, all secondary antibodies above were diluted 1 : 5000) for 90 min at room temperature. After the membranes were washed with TBST again, the immune complexes were detected by enhanced chemiluminescence reagents. The images were quantified using a Bio-Rad ChemiDoc EQ densitometer and Bio-Rad Quantity One software (Bio-Rad Laboratories, Hercules, CA, USA).

### 2.8. Monodansylcadaverine (MDC) Staining

MDC is a phospholipid-specific marker for lysosomal activity and fused autolysosomes, which appear as distinct dot-like structures. MDC is widely used for autophagic vacuole detection. At 30 min after HSYA-SDT, cells were incubated with MDC (50 *μ*M, Cayman Chemical Co., USA) for 30 min at 37°C in the dark. After the cells were labeled, they were washed carefully with PBS, and the images were observed by fluorescence microscopy at 372 nm excitation and 456 nm emission wavelengths.

### 2.9. Immunofluorescence

The cells were seeded in 20 mm glass-bottom cell culture dishes. After treatment, the cells were fixed with 4% paraformaldehyde for 30 min and permeabilized with 0.1% Triton X-100 for 20 min. Then, cells were blocked with 3% bovine serum albumin (BSA) for 30 min at room temperature and incubated with anti-LC3 antibody (1 : 100, Santa Cruz Biotechnology, Inc., USA) overnight at 4°C. The cells were washed twice with PBS and incubated with the corresponding secondary antibody for 1 h at 37°C. Nuclei were stained with DAPI for 5 min at 37°C in the dark. After the cells were washed twice with PBS, they were examined by laser-scanning confocal microscopy (LSCM; LSCM 510 Meta; Zeiss, Gottingen, Germany).

### 2.10. Transfection of Small Interfering RNA (siRNA)

Knockdown of Atg5 or mTOR in macrophages was performed using siRNA oligonucleotides targeting the cDNA sequence of human Atg5 and mTOR according to the manufacturer's protocols (Invitrogen Life Technologies, Inc., Carlsbad, CA, USA). The cells were incubated in 35 mm dishes and transfected with siRNA. An irrelevant 21 nucleotide siRNA (GenePharma Co., Ltd., Shanghai, China) was used as the negative Control. First, 10 *μ*L of siRNA (20 *μ*M) was mixed with 100 *μ*L Opti-MEM media (Invitrogen Life Technologies, Inc., Carlsbad, CA, USA). In addition, 10 *μ*L X-tremeGene Transfection Reagent (Roche, Basel, Switzerland) was mixed with 100 *μ*L Opti-MEM. Then, the two samples were mixed together and combined for 20 min. The mixtures were added to each Petri dish containing fresh medium and incubated for 6 h at 37°C in the dark. Medium was then changed to antibiotic-free FBS-loaded RPMI 1640 medium for 48 h at 37°C in the dark.

*Atg5 siRNA Duplexes*

S1 (sense, GCAGUGGCUGAGUGAACAUTT, antisense, AUGUUCACUCAGCCACUGCTT)
S2 (sense, CCAUCAAUCGGAAACUCAUTT, antisense, AUGAGUUUCCGAUUGAUGGTT)
S3 (sense: GCUUCGAGAUGUGUGGUUUTT; antisense: AAACCACACAUCUCGAAGCTT)

*mTOR siRNA Duplexes*

S1 (sense, CCACCCGAAUUGGCAGAUUTT, antisense, AAUCUGCCAAUUCGGGUGGTT)
S2 (sense, GCAAAGAUCUCAUGGGCUUTT, antisense, AAGCCCAUGAGAUCUUUGCTT)
S3 (sense, CCAAGAUACCAUGAACCAUTT, antisense, AUGGUUCAUGGUAUCUUGGTT)

### 2.11. Enzyme Linked Immunosorbent Assay (ELISA)

For detection of the cytokines released by THP-1 macrophages in vitro, the cell supernatant was collected after treatment, and TNF-*α*, IL-12, and IL-1*β* were measured by ELISA kits (Elabscience Biotechnology Co., Ltd., Wuhan, China) following the manufacturer's protocols.

### 2.12. Statistical Analysis

All experiments were replicated independently at least three times. The data were analyzed using one-way analysis of variance (ANOVA) and are presented as the mean ± standard deviation (SD). Statistical significance was defined as *p* < 0.05.

## 3. Results

### 3.1. Physical Optics Characterization of HSYA and SDT Parameter Selection

**Figure 1:**
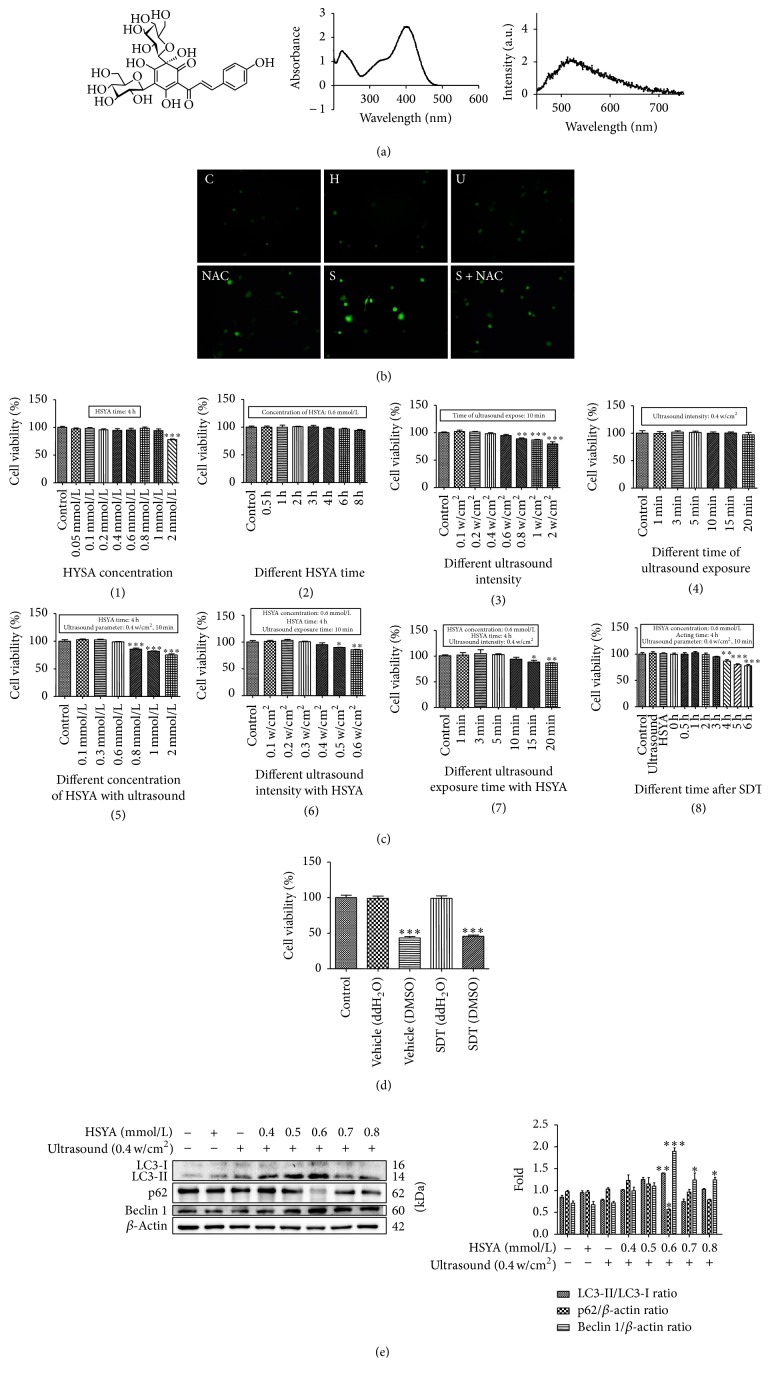
Physical optics characterization of HSYA and SDT parameter selection. (a) The chemical structure, absorption spectrum, and fluorescence emission spectrum of HSYA (dissolved in ddH_2_O). (b) Intracellular ROS generation of THP-1 macrophages was measured by DCFH-DA staining (scale bar: 50 *μ*m). (c) Effects of HSYA with or without ultrasound irradiation on the viability of THP-1 macrophages. With (1) different concentrations of HSYA, (2) different HSYA exposure time, (3) different ultrasound intensity, (4) different ultrasound exposure time, (5) HSYA-SDT (different concentrations) (0.4 W/cm^2^ ultrasound irradiation), (6) HSYA-SDT (0.6 mmol/L) (different ultrasound intensity), (7) HSYA-SDT (0.6 mmol/L) (different ultrasound exposure time), and (8) HSYA-SDT (0.6 mmol/L) (different time after SDT), cell viabilities were analyzed by CCK-8 assays. (d) Effects of different solvents of HSYA with or without ultrasound on the viability of THP-1 macrophages. Cell viabilities were analyzed by CCK-8 assays. (e) LC3-Ι, LC3-ΙΙ, p62, and beclin 1 protein expression was analyzed by Western blots post-HSYA-SDT (different concentrations), and quantifications of the LC3-ΙΙ/LC3-Ι ratio, p62, and beclin 1 are shown. All data are mean ± standard error (*n* = 5). ^*∗*^*p* < 0.05, ^*∗∗*^*p* < 0.01, and ^*∗∗∗*^*p* < 0.001 versus Control.

Figure 1(a) shows the chemical structure, absorption spectrum, and fluorescence emission spectrum of HSYA. ROS generation is considered the major mechanism of SDT; therefore, we measured the ROS production using the intracellular ROS probe DCFH-DA. The green fluorescence increased slightly in the HSYA alone and ultrasound alone groups, whereas it increased substantially in the SDT group. The increase in ROS production could be blocked by the ROS scavenger NAC (Figure 1(b)). Thus, we showed that HSYA could be used as a sonosensitizer.

To select the optimal conditions for HSYA-SDT, we determined the cell viabilities after different treatments using CCK-8 assays. Ultrasound intensity and exposure time, HSYA concentration, and time were optimized to ensure that the ultrasound, HSYA alone, or a combination treatment did not affect cell survival. The data showed that the survival rate decreased significantly at a concentration of 2 mmol/L HSYA concentration (Figure 1(c)(1)). Cell viability was maintained during 0.6 mmol/L HSYA with increasing incubation time until 8 h (Figure 1(c)(2)) and also maintained until 0.8 w/cm^2^ of ultrasound alone (Figure 1(c)(3)) and remained stable at 0.4 w/cm^2^ ultrasound irradiation with increasing exposure time (Figure 1(c)(4)). When we used the HSYA together with ultrasound exposure, cell viability was maintained until 0.6 mmol/L HSYA concentration, 0.4 w/cm^2^, and 10 min ultrasound irradiation (Figure 1(c)((5)–(7))). Thus, for safety, we chose a 10 min exposure time, 0.4 w/cm^2^ as the ultrasound parameters, and a HSYA concentration less than 0.8 mmol/L. Cell viability was maintained stably until 4 h after SDT (Figure 1(c)(8)). Moreover, we also used dimethyl sulfoxide (DMSO) for the HSYA solution and compared it to ddH_2_O. As shown in Figure 1(d), DMSO as a solvent could lead to significant cytotoxicity regardless of the occurrence of HSYA-SDT. Thus, we chose ddH_2_O to be the solvent.

To determine the effect of HSYA-SDT on autophagy, we examined the expression of autophagy-related proteins by Western blots. LC3 processing is a classical marker of autophagy, and the amount of LC3-II levels is correlated with the formation of autophagic vacuoles [32]. Beclin 1 is an autophagy-related gene and a critical factor that affects the induction of autophagy [33]. Additionally, p62 can bind to the autophagosome membrane for degradation of target aggregates in autophagosomes [34]. LC3-II, beclin 1, and p62 degradation increased significantly following the combination of ultrasound and 0.6 mmol/L HSYA (Figure 1(e)). Accordingly, we chose 0.6 mmol/L HSYA in HSYA-SDT in THP-1 macrophages.

### 3.2. HSYA-SDT Induced Autophagy in THP-1 Macrophages

**Figure 2:**
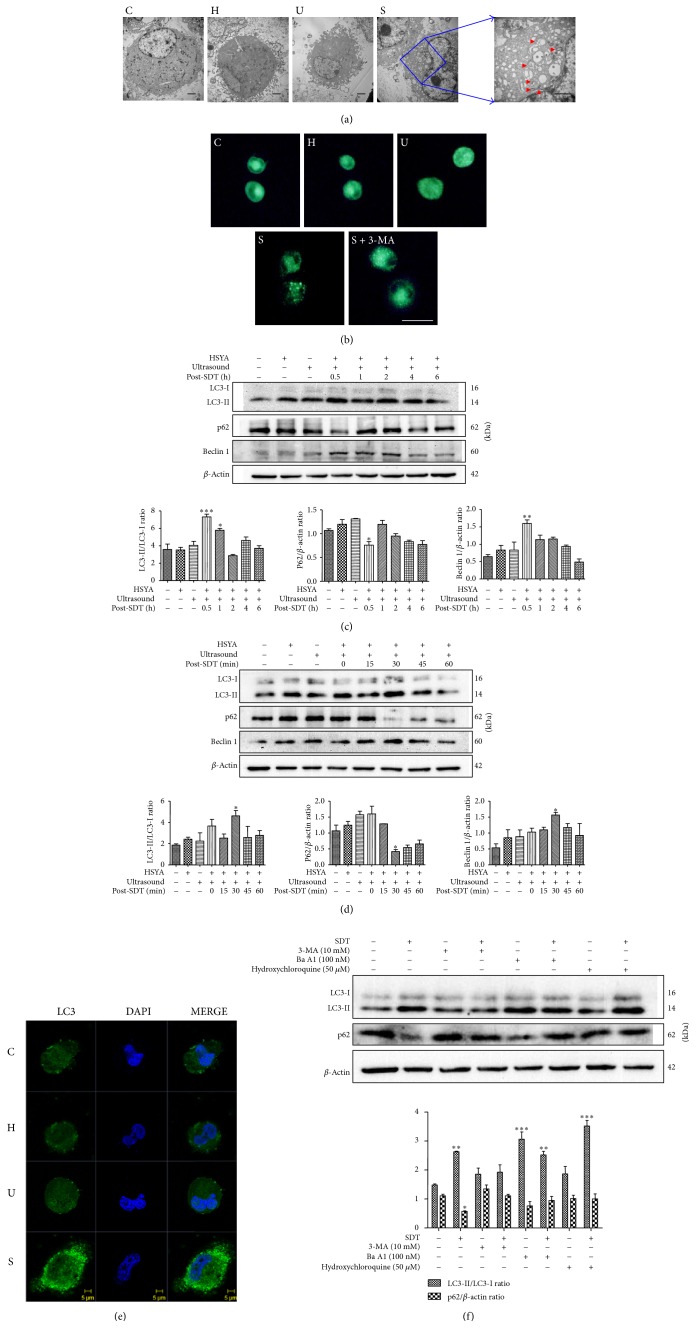
HSYA-SDT induced autophagy in THP-1 macrophages. (a) Morphological alterations of THP-1 macrophages were observed using TEM. Red arrows indicate the autophagosomes (scale bar: 2 *μ*m). (b) Autophagic vacuoles induced by different treatments were observed using MDC staining of HSYA-SDT-treated cells with or without 3-MA (10 mM) pretreatment. THP-1 macrophages were incubated with MDC (50 *μ*M) for 30 min at 30 min after HSYA-SDT (scale bar: 20 *μ*m). (c) LC3-Ι, LC3-ΙΙ, p62, and beclin 1 protein expression was analyzed by Western blots at different time points after HSYA-SDT, and quantifications of the LC3-ΙΙ/LC3-Ι ratio, p62, and beclin 1 are shown. (d) LC3-Ι, LC3-ΙΙ, p62, and beclin 1 protein expression was analyzed by Western blots at different time points after HSYA-SDT, and quantifications of the LC3-ΙΙ/LC3-Ι ratio, p62, and beclin 1 are shown. (e) Cells were stained with an anti-LC3 antibody and DAPI at 30 min after HSYA-SDT (scale bar: 5 *μ*m). (f) The effect of 3-MA, hydroxychloroquine, and ba A1 with or without HSYA-SDT on the expression levels of the autophagy-related proteins p62 and LC3 and quantifications of the LC3-ΙΙ/LC3-Ι ratio and p62 are shown. All data are mean ± standard error (*n* = 5). ^*∗*^*p* < 0.05, ^*∗∗*^*p* < 0.01, and ^*∗∗∗*^*p* < 0.001 versus Control.

Macrophage ultrastructure was observed using TEM to further assess the autophagy-inducing effects of HSYA-SDT. Cells treated with HSYA-SDT had autophagosomes with distinct double membranes or myelin figures, while these structures were rare in the Control groups (Figure 2(a)). MDC staining indicated an increase in MDC-labeled vesicles in the HSYA-SDT group, while in the other groups, diffuse staining with minimal puncta was observed (Figure 2(b)). Thus, autophagic vacuoles formation was induced following HSYA-SDT. The conversion of LC3-I to LC3-II and expression of p62 and beclin 1 at various time points post-HSYA-SDT were examined by Western blots. First, we analyzed these variables at 0.5 h, 1 h, 2 h, 4 h, and 6 h; LC3-II and beclin 1 increased significantly concomitant with decreased p62 at 0.5 h after HSYA-SDT (Figure 2(c)). Then, we narrowed the range to 0 min, 15 min, 30 min, 45 min, and 60 min and found that increases in LC3-II and beclin 1 and degradation of p62 were the strongest at 30 min after HSYA-SDT (Figure 2(d)). Hence, 30 min after HSYA-SDT was the peak time for induction of autophagy in THP-1 macrophages. Additionally, we evaluated the relative fluorescence of LC3 using confocal laser-scanning microscopy. HSYA-SDT-treated macrophages showed a greater number of LC3-stained punctate spots than those in other groups (Figure 2(e)). These results indicated that HSYA-SDT could induce autophagy in THP-1 macrophages and the peak time was 30 min after HSYA-SDT.

We used 3-MA, a common autophagy inhibitor, and hydroxychloroquine, and ba A1, inhibitors of autophagy that block LC3-II degradation. HSYA-SDT-induced LC3 conversion and p62 degradation were suppressed by 3-MA, and both LC3-II and p62 levels increased significantly after pretreatment with hydroxychloroquine and ba A1 (Figure 2(f)), which indicated an intact autophagic flux following HSYA-SDT.

### 3.3. HSYA-SDT Induced Autophagy by Inhibiting the PI3K/AKT/mTOR Signaling Pathway in THP-1 Macrophages

**Figure 3:**
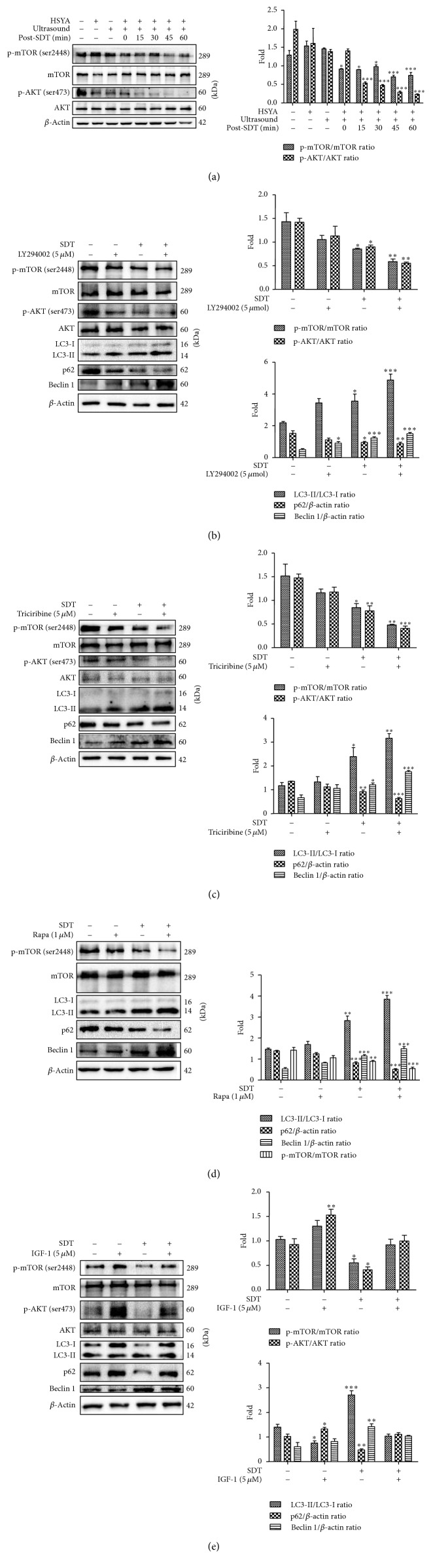
HSYA-SDT induced autophagy by inhibiting the PI3K/AKT/mTOR signaling pathway in THP-1 macrophages. (a) mTOR, p-mTOR (Ser 2448), AKT, and p-AKT (Ser 473) protein expression was analyzed by Western blots at different time points after HSYA-SDT, and quantifications of the p-mTOR/mTOR ratio and p-AKT/AKT ratio are shown. (b) The effect of LY294002 on the expression levels of mTOR, p-mTOR (Ser 2448), AKT, p-AKT (Ser 473), LC3-Ι, LC3-ΙΙ, p62, and beclin 1 protein at 30 min after SDT, and quantifications of the proteins above are shown. (c) The effect of triciribine on the expression levels of mTOR, p-mTOR (Ser 2448), AKT, p-AKT (Ser 473), LC3-Ι, LC3-ΙΙ, p62, and beclin 1 protein at 30 min after SDT, and quantifications of the proteins above are shown. (d) The effect of rapamycin on the expression levels of mTOR, p-mTOR (Ser 2448), LC3-Ι, LC3-ΙΙ, p62, and beclin 1 protein at 30 min after SDT, and quantifications of the proteins above were shown. (e) The effect of IGF-1 on the expression levels of mTOR, p-mTOR (Ser 2448), AKT, p-AKT (Ser 473), LC3-Ι, LC3-ΙΙ, p62, and beclin 1 protein at 30 min after SDT, and quantifications of the proteins above were shown. All data are mean ± standard error (*n* = 5). ^*∗*^*p* < 0.05, ^*∗∗*^*p* < 0.01, and ^*∗∗∗*^*p* < 0.001 versus Control.

mTOR is a signaling molecule of the PI3K/AKT/mTOR pathway and closely associated with the inhibition of autophagy [35, 36]. The total and phosphorylated levels of AKT and mTOR were measured to determine whether the PI3K/AKT/mTOR pathway was involved in HSYA-SDT-induced autophagy. As shown in Figure 3(a), the AKT phosphorylation at Ser473 and mTOR phosphorylation at Ser2448 decreased significantly after HSYA-SDT. Additionally, significant decreases in the levels of p-AKT, p-mTOR, and p62 and significant increases in LC3-II and beclin 1 were observed after pretreatment of the cells with the PI3K inhibitors LY294002 (Figure 3(b)), the AKT inhibitors triciribine (Figure 3(c)), and the mTOR inhibitors rapamycin (Figure 3(d)) prior to HSYA-SDT treatment, respectively. The levels of AKT phosphorylation at Ser473 and mTOR phosphorylation at Ser2448 increased significantly after pretreatment with the PI3K agonist IGF-1 (Figure 3(e)). These results indicated that p-AKT and p-mTOR were key factors involved in HSYA-SDT-induced autophagy in THP-1 macrophages.

### 3.4. Knockdown of Atg5 or mTOR Had Different Effects on HSYA-SDT-Induced Autophagy in THP-1 Macrophages

**Figure 4:**
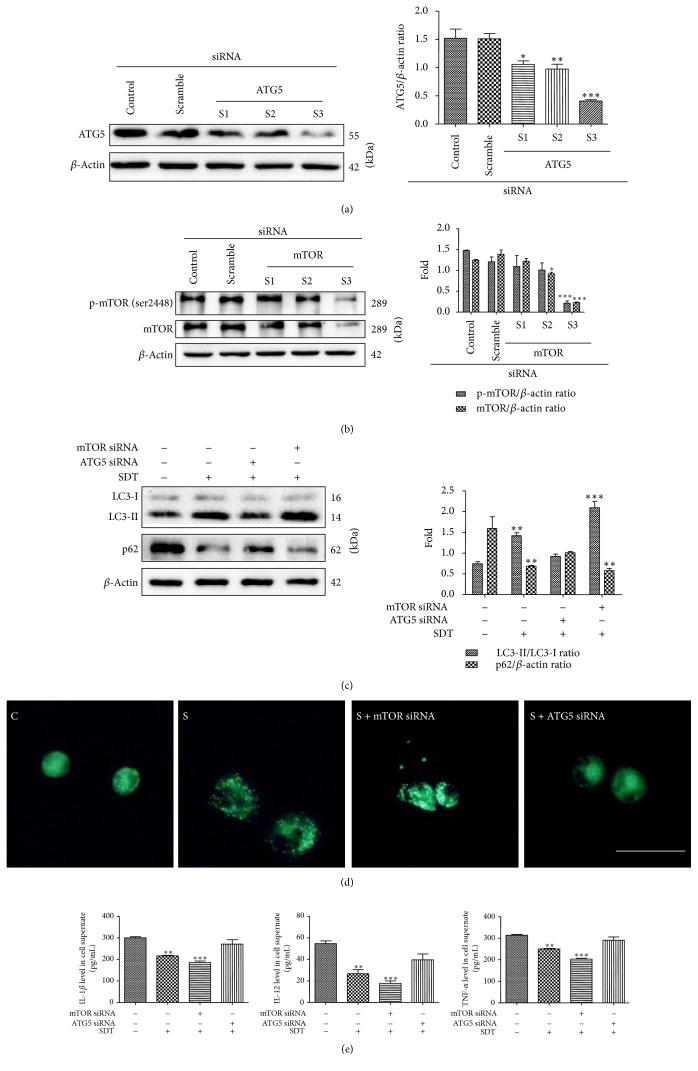
Knockdown of Atg5 or mTOR had different effects on HSYA-SDT-induced autophagy in THP-1 macrophages. (a) Representative Western blots and quantification of Atg5 following siRNA treatment are shown. (b) Representative Western blots and quantification of mTOR and p-mTOR following siRNA treatment are shown. (c) LC3-Ι, LC3-ΙΙ, and p62 protein expression was analyzed by Western blots post-HSYA-SDT following Atg5 or mTOR siRNA treatment, and quantifications of the LC3-ΙΙ/LC3-Ι ratio and p62 are shown. (d) Autophagic vacuoles were analyzed using MDC staining in HSYA-SDT-treated cells following Atg5 or mTOR siRNA treatment (scale bar, 20 *μ*m). (e) ELISAs of the inflammatory factors TNF-*α*, IL-12, and IL-1*β* secreted by THP-1 macrophages following Atg5 or mTOR siRNA treatment. All data are mean ± standard error (*n* = 5). ^*∗*^*p* < 0.05, ^*∗∗*^*p* < 0.01, and ^*∗∗∗*^*p* < 0.001 versus Control.

To further examine the autophagy induced by HSYA-SDT, we knocked down the autophagy-related proteins Atg5 (Figure 4(a)) and mTOR (Figure 4(b)) using siRNA. HSYA-SDT-induced LC3 conversion and p62 degradation were suppressed by knockdown of Atg5, but LC3-II and p62 degradation increased significantly after knockdown of mTOR (Figure 4(c)). MDC staining showed an increase in MDC-labeled vesicles in the mTOR knockdown group and a decrease in the Atg5 knockdown group compared with that of the HSYA-SDT group (Figure 4(d)). As shown in Figure 4(e), ELISAs showed a decrease of the inflammatory factors TNF-*α*, IL-12, and IL-1*β* in cell supernate in the mTOR knockdown group but an increase in the Atg5 knockdown group compared to that in the HSYA-SDT group. These results suggested that HSYA-SDT-induced autophagy and inflammation inhibition could be blocked by knockdown of Atg5 but was activated by knockdown of mTOR.

### 3.5. HSYA-SDT Inhibited Expression and Secretion of Inflammatory Factors in THP-1 Macrophages

**Figure 5:**
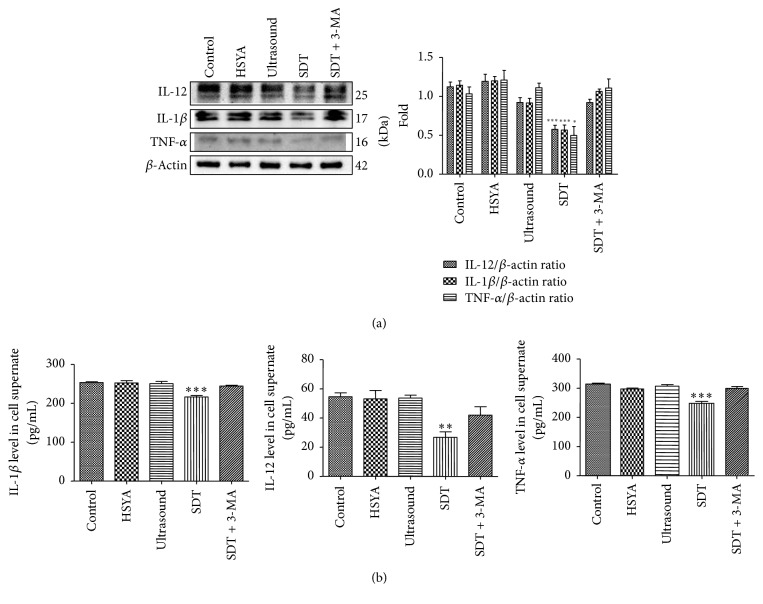
HSYA-SDT could inhibit expression of inflammatory factors in THP-1 macrophages. (a) Protein extracts from untreated cells (Control) and cells treated with HSYA, ultrasound, HSYA-SDT, or 3-MA prior to HSYA-SDT were analyzed by Western blots to detect TNF-*α*, IL-12, and IL-1*β*. Quantifications of protein expression are also provided. (b) ELISAs of the inflammatory factors TNF-*α*, IL-12, and IL-1*β* secreted by THP-1 macrophages with different treatments. All data are mean ± standard error (*n* = 5). ^*∗*^*p* < 0.05, ^*∗∗*^*p* < 0.01, and ^*∗∗∗*^*p* < 0.001 versus Control.

To further determine whether autophagy induced by HSYA-SDT plays an important role in inhibiting inflammation, we performed Western blots and ELISAs of THP-1 macrophages to detect the expression and secretion of the inflammatory factors TNF-*α*, IL-12, and IL-1*β*. As shown in Figure 5(a), TNF-*α*, IL-12, and IL-1*β* levels decreased significantly after HSYA-SDT. Moreover, ELISAs also confirmed the decrease in the inflammatory factors TNF-*α*, IL-12, and IL-1*β* in cell supernatant of the HSYA-SDT group compared to that of the Control group, HSYA alone group, ultrasound alone group, and 3-MA pretreatment group (Figure 5(b)). These results suggested that HSYA-SDT could inhibit expression and secretion of inflammatory factors in THP-1 macrophages through activation of autophagy.

### 3.6. Autophagy Triggered by HSYA-SDT through the PI3K/Akt/mTOR Pathway and the Inhibition of Inflammatory Factors Were Suppressed by the ROS Scavenger NAC

**Figure 6:**
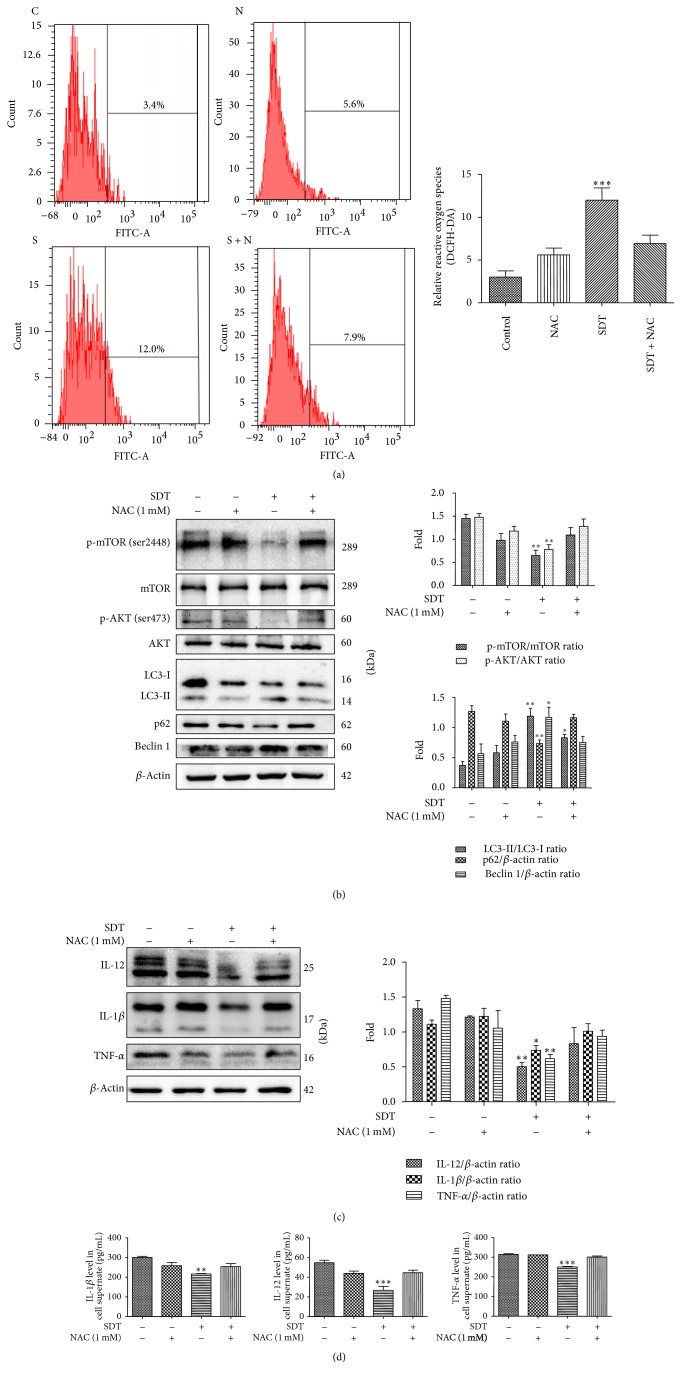
Autophagy triggered by HSYA-SDT through the PI3K/Akt/mTOR pathway and the inhibition of inflammatory factors were suppressed by the ROS scavenger NAC. (a) The relative fluorescence intensity of ROS generation detected in THP-1 macrophages with or without pretreatment with NAC was measured by flow cytometry. (b) The effect of NAC on the expression levels of mTOR, p-mTOR (Ser 2448), AKT, p-AKT (Ser 473), LC3-Ι, LC3-ΙΙ, p62, and beclin 1 at 30 min after SDT was determined, and quantifications of the proteins above are shown. (c) Protein extracts from untreated cells (Control) and cells treated with NAC, HSYA-SDT, or NAC prior to HSYA-SDT were analyzed by Western blots to detect TNF-*α*, IL-12, and IL-1*β*. Quantifications of protein expression are also provided. (d) ELISAs of the inflammatory factors TNF-*α*, IL-12, and IL-1*β* secreted by THP-1 macrophages with or without pretreatment by NAC. All data are mean ± standard error (*n* = 5). ^*∗*^*p* < 0.05, ^*∗∗*^*p* < 0.01, and ^*∗∗∗*^*p* < 0.001 versus Control.

Production of ROS was analyzed using DCFH-DA staining by flow cytometry. The ROS levels increased following HSYA-SDT compared to those of the Control group, NAC alone group, and NAC pretreatment with HSYA-SDT group (Figure 6(a)). These data demonstrated that HSYA-SDT-induced ROS production could be effectively blocked by the ROS scavenger NAC.

To investigate whether the autophagy induced by HSYA-SDT was associated with the generation of ROS, we examined p62 degradation, LC3 conversion, beclin 1 expression, and the total and phosphorylated levels of AKT and mTOR following pretreatment with NAC by Western blots. As shown in Figure 6(b), significant decreases in the levels of p-AKT, p-mTOR, and p62 and significant increases in LC3-II and beclin 1 post-HSYA-SDT were inhibited by NAC. Additionally, pretreatment with NAC after HSYA-SDT increased the expression and secretion of TNF-*α*, IL-12, and IL-1*β* compared with those of the HSYA-SDT alone group (Figures 6(c) and 6(d)). Thus, we showed that HSYA-SDT induced autophagy and inhibited inflammatory factors through ROS production.

### 3.7. Schematic Diagram of the Proposed Mechanism Underlying HSYA-SDT-Induced Autophagy and Inflammation Inhibition in THP-1 Macrophages

**Figure 7:**
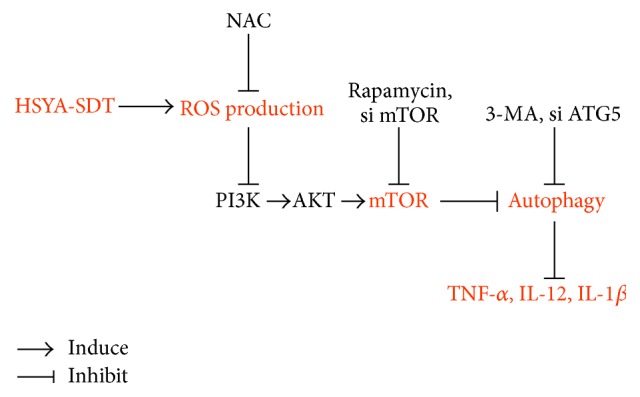
Schematic diagram of the proposed mechanism underlying HSYA-SDT-induced autophagy and inflammation inhibition in THP-1 macrophages. HSYA-mediated SDT induces an autophagic response through the PI3K/Akt/mTOR signaling pathway mediated by ROS, thereby inhibiting inflammation.

Taken together, as illustrated schematically in Figure 7, findings from the present study have demonstrated that HSYA-SDT induces an autophagic response via the PI3K/Akt/mTOR signaling pathway and inhibits inflammation by ROS in THP-1 macrophages.

## 4. Discussion

In the initiation and development of atherosclerosis, monocyte-derived macrophages participate in inflammatory responses by secreting various inflammatory factors [37]. In the present study, we selected THP-1 macrophages as a cell model and IL-1*β*, IL-12, and TNF-*α* as typical inflammatory factors.

The generation of ROS is considered to be the most important mechanism of SDT [25, 38, 39]. In the present study, a significant increase of ROS generation was detected in the HSYA-SDT group compared to that of the Control groups. Moreover, scavenging of ROS significantly inhibited autophagy, indicating that HSYA-SDT-induced ROS generation activated autophagy in THP-1 macrophages. These results are consistent with those of other SDT-related studies [25, 38, 39]. Blockage of ROS could also diminish the HSYA-SDT-induced inflammatory inhibition in our study, in contrast to several previous reports regarding the proinflammatory function of ROS, such as Lee and Yang [40]. To generate an optimal ROS level, we assayed HSYA-SDT parameters in THP-1 macrophages based on cell safety and the ability to induce autophagy. However, ROS overproduction was observed in other studies [40]. Therefore, varying ROS levels may account for the difference in results.

The rapidly expanding body of literature on SDT has shown that the development of sonosensitizers is one of the most essential factors in this treatment. In recent years, our group has identified sonosensitizers derived from extracts of Chinese herbs, including hypericin, curcumin, and hydroxyl acetylated curcumin [2, 20, 21]. However, further animal experiments and clinical translation of these sonosensitizers are limited by their lipophilic properties and rare application in the clinic. HSYA is isolated from the hydrophilic fraction of a traditional Chinese herbal medicine, safflower plant. It is generally used in the clinical treatment of ischemic diseases, trauma, cardiovascular diseases, and cerebrovascular diseases by intravenous injection [30, 31]. Thus, HSYA has higher water-solubility and safety than those of the above sonosensitizers. In the present study, we investigated whether HSYA could be used as a sonosensitizer.

Autophagy is a complex intracellular process for cytoplasmic component delivery [8]. In the present study, expression of autophagy-related proteins, MDC staining, and TEM data showed induction of autophagy in the HSYA-SDT group, but application of HSYA or ultrasound alone did not have these effects. Moreover, the autophagy induced by HSYA-SDT changed in a time-dependent manner and peaked at 30 min posttreatment, which is similar to the induction of autophagy following protoporphyrin IX-SDT of K562 cells as reported by Su et al. [41].

mTOR is a serine/threonine protein kinase that plays an important role in cell survival, proliferation, and metabolism [42, 43]. The PI3K/AKT/mTOR signaling pathway is a classical autophagic pathway. In the present study, AKT phosphorylation at Ser473 and mTOR phosphorylation at Ser2448 decreased significantly after HSYA-SDT of THP-1 macrophages. The inhibitors of this pathway, mTOR siRNA, reduced AKT and mTOR phosphorylation. Moreover, blockage of ROS reversed the HSYA-SDT-induced decrease in AKT and mTOR phosphorylation. These results indicated that suppression of the PI3K/AKT/mTOR signaling pathway was involved in the HSYA-SDT-induced autophagy via ROS production.

In atherosclerosis, activation of inflammation can lead to protease secretion, tissue destruction, and plaque rupture [44–46]. IL-1*β*, IL-12, and TNF-*α* are typical inflammatory factors, and in the present study, the expression and secretion of these inflammatory factors were significantly reduced after HSYA-SDT. These changes were reversed by autophagy inhibition and ROS blockage. These results indicated that autophagy induced by HSYA-SDT inhibited inflammation via ROS generation in THP-1 macrophages.

In conclusion, our study suggests that HSYA-mediated SDT induces an autophagic response through suppression of the PI3K/Akt/mTOR signaling pathway and inhibits inflammation by ROS in THP-1 macrophages. Further animal studies should be performed to develop SDT as a novel treatment for atherosclerosis.
